# Interspecies Papillomavirus Type Infection and a Novel Papillomavirus Type in Red Ruffed Lemurs (*Varecia rubra*)

**DOI:** 10.3390/v16010037

**Published:** 2023-12-25

**Authors:** Elise N. Paietta, Simona Kraberger, Melanie Regney, Joy M. Custer, Erin Ehmke, Anne D. Yoder, Arvind Varsani

**Affiliations:** 1 Department of Biology, Duke University, Durham, NC 27708, USA; anne.yoder@duke.edu; 2 The Biodesign Center for Fundamental and Applied Microbiomics, Center for Evolution and Medicine, School of Life Sciences, Arizona State University, Tempe, AZ 85287, USA; simona.kraberger@asu.edu (S.K.); mregney@asu.edu (M.R.); joy.m.custer@gmail.com (J.M.C.); 3 Duke Lemur Center, Durham, NC 27705, USA; erin.ehmke@duke.edu; 4 Structural Biology Research Unit, Department of Integrative Biomedical Sciences, University of Cape Town, Cape Town 7925, South Africa

**Keywords:** Papillomaviridae, Varecia rubra, lemur

## Abstract

The *Papillomaviridae* are a family of vertebrate-infecting viruses of oncogenic potential generally thought to be host species- and tissue-specific. Despite their phylogenetic relatedness to humans, there is a scarcity of data on papillomaviruses (PVs) in speciose non-human primate lineages, particularly the lemuriform primates. *Varecia variegata* (black-and-white ruffed lemurs) and *Varecia rubra* (red ruffed lemurs), two closely related species comprising the *Varecia* genus, are critically endangered with large global captive populations. Varecia variegata papillomavirus (VavPV) types −1 and −2, the first PVs in lemurs with a fully identified genome, were previously characterized from captive *V. variegata* saliva. To build upon this discovery, saliva samples were collected from captive *V. rubra* with the following aims: (1) to identify PVs shared between *V. variegata* and *V. rubra* and (2) to characterize novel PVs in *V. rubra* to better understand PV diversity in the lemuriform primates. Three complete PV genomes were determined from *V. rubra* samples. Two of these PV genomes share 98% L1 nucleotide identity with VavPV2, denoting interspecies infection of *V. rubra* by VavPV2. This work represents the first reported case of interspecies PV infection amongst the strepsirrhine primates. The third PV genome shares <68% L1 nucleotide identity with that of all PVs. Thus, it represents a new PV species and has been named Varecia rubra papillomavirus 1 (VarPV1). VavPV1, VavPV2, and VarPV1 form a new clade within the *Papillomaviridae* family, likely representing a novel genus. Future work diversifying sample collection (i.e., lemur host species from multiple genera, sample type, geographic location, and wild populations) is likely to uncover a world of diverse lemur PVs.

## 1. Introduction

Papillomaviruses (PVs) are double-stranded DNA viruses with icosahedral capsids that infect diverse vertebrates, including mammal, avian, fish, and reptile species [1,2]. Depending on the PV type, infected hosts can experience a variety of disease outcomes ranging from asymptomatic cases—the majority of PV infections—to benign papillomas and even progressing to invasive cancer [3,4,5,6]. PV genomes are ~6–8 kb in size and encode at least four proteins (i.e., two early proteins—E1, E2, and two late proteins—L1, L2) with high conservation of the L1 capsid protein across PVs [1,7]. PVs have co-evolved with their hosts and are generally recognized as host species- and tissue-specific [4,8]. However, the assumption that PVs are primarily host species-specific is being increasingly challenged by the detection of several PV types in species other than their host species [9]. While host–pathogen co-evolution, intra-host duplication, adaptive radiation, and recombination have been found to be drivers of PV evolution, mounting evidence of interspecies infection suggests that host-switching could also serve as a crucial evolutionary force in the history of PVs [9,10,11,12,13,14].

Cross-species PV infections leading to tumor disease are described for several PVs, especially those in the genus *Deltapapillomavirus*, including bovine papillomavirus (BPV) types −1, −2, or −13 in equine sarcoids [15,16,17], BPV-1 in captive tapir sarcoids [18], BPV-2 in bladder tumors of water buffalo [19], BPV-1 in cutaneous and perivulvar fibropapillomas in water buffalo [20], and BPV-14 in feline sarcoids in domestic cats and captive African lions [21,22]. Cross-species experimental inoculation with BPV1 has been shown to induce fibromas and fibrosarcomas in hamsters and mice [23,24,25] and oral papillomas in domestic dogs from coyote oral papillomavirus [26]. Overall, BPVs, in particular, have been repeatedly observed to have interspecies hosts with pathogenic effects on new host species. This may in part be due to the tissue tropism of PVs in the genus *Deltapapillomavirus,* as they can infect dermal fibroblasts [27,28].

**Figure 1:**
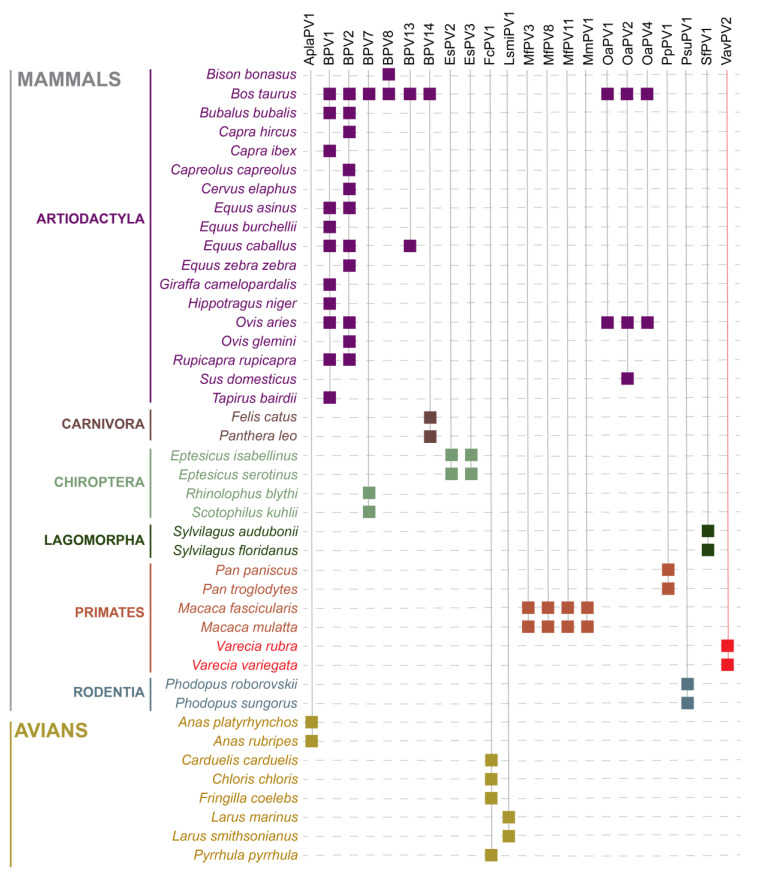
Summary of evidence available throughout the literature and NCBI Virus (https://www.ncbi.nlm.nih.gov/labs/virus/vssi/ (accessed on 1 September 2023)) of naturally occurring interspecies infection in mammalian and avian orders. PVs are connected via solid vertical lines to the animal species they have been found to infect. Abbreviations and references for PV types are as follows: Anas platyrhynchos papillomavirus 1 (AplaPV1) [10], bovine papillomavirus types −1, −2, −7, −8, −13, −14 (BPV1, BPV2, BPV7, BPV8, BPV13, BPV14) [12,16,18,19,20,21,22,28,29,30,31,32,33,39,41,42,43,44,45], Eptesicus serotinus papillomavirus types −2, −3 (EsPV2, EsPV3) [13], Fringilla coelebs papillomavirus 1 (FcPV1) [34], Larus smithsonianus papillomavirus 1 (LsmiPV1) [10], Macaca fascicularis papillomavirus types −3, −8, −11 (MfPV3, MfPV8, MfPV11) [38], Macaca mulatta papillomavirus 1 (MmPV1) [38], Ovis aries papillomavirus type −1, −2, −4 (OaPV1, OaPV2, OaPV4) [27,40,46], Pan paniscus papillomavirus 1 (PpPV1) [35,36], Phodopus sungorus papillomavirus type 1 (PsuPV1) [9], Sylvilagus floridanus papillomavirus 1 (SfPV1) [37], Varecia variegata papillomavirus 2 (VavPV2).

Beyond BPVs, naturally occurring interspecies PV infection has been seen across numerous mammalian orders (Artiodactyla, Carnivora, Chiroptera, Lagomorpha, Primates, and Rodentia) and avian orders (Anseriformes, Charadriiformes, and Passeriformes) (Figure 1) [9,10,12,13,16,18,19,20,21,22,27,28,29,30,31,32,33,34,35,36,37,38,39,40,41,42,43,44,45,46]. Interspecies PV infection in non-human primates has only been detected in very closely phylogenetically related species in the same genus. Four alphapapillomavirus types, Macaca fascicularis papillomavirus (MfPV) types −1, −8, −11, and Macaca mulatta papillomavirus (MmPV) −1, have been detected across rhesus macaques (*Macaca mulatta*) and cynomolgus macaques (*Macaca fascicularis*). Additionally, Pan paniscus papillomavirus 1 (PpPV1) was isolated from both chimpanzees (*Pan troglodytes*) and bonobos (*Pan paniscus*), although it is unclear which species served as the first host of PpPV1 [35].

Only 16 out of >390 non-human primate species have been screened for PVs (Table 1). Acknowledging ongoing taxonomic adjustments across all non-human primate lineages, the New World monkeys (>100 species), Old World monkeys (>130 species), and lemurs (>100 species) are the most speciose non-human primate lineages [47,48,49,50]. For understanding PV diversity in these vast lineages, just 8 complete PV genomes across 5 New World monkey species are available on NCBI, while 23 complete PV genomes across 6 Old World monkey species are currently available (Table 1). Further, despite lemuriforms comprising ~20% of primate species, only three complete genomes of PVs in lemurs from just one host species had been characterized prior to this study.

We previously identified two PV types, Varecia variegata papillomavirus (VavPV) −1 and −2, from captive black-and-white ruffed lemur (*Varecia variegata variegata*) saliva samples, providing the first complete genomes of PVs in the lemuriform primates [51]. VavPV1 and VavPV2 share <64% L1 identity with one another and <66% L1 identity with all other PV L1 sequences. VavPV1 and −2 formed a distinct clade within the *Firstpapillomavirinae* sub-family and likely represent a novel genus [51].

As interspecies PV type infection in primates has been detected in sister species, we obtained saliva samples from red ruffed lemurs (*Varecia rubra*), the only other species within the *Varecia* genus. Our primary goals were (1) to identify PVs shared between *V. variegata* and *V. rubra* and (2) to identify any new PVs in *V. rubra* to better understand PV diversity in the lemuriform primates using viral metagenomics. Occurring in the eastern rainforests of Madagascar, *V. variegata* and *V. rubra* are diurnal, frugivorous lemurs vital to their ecosystems as seed dispersers and pollinators [47]. Both are classified as critically endangered and, thus, have extensive global captive populations [52,53]. The work presented here is relevant for the health of captive *Varecia* populations and for forming a baseline of our knowledge of lemur PVs from which future comparisons between captive and wild *Varecia* PVs will be possible.

## 2. Materials and Methods

To characterize additional novel lemur PVs and to identify potential interspecies infection by PV types, two saliva samples from captive red ruffed lemurs (*Varecia rubra*) were collected at the Duke Lemur Center in Durham (NC, USA). The sampled lemurs appeared healthy with no apparent symptoms and continued to be monitored by veterinary staff. *V. rubra* saliva samples were collected under IACUC #A161-21-08 in August–September 2022. Saliva was obtained by allowing the lemurs to chew on a SalivaBio Children’s Swab (Salivametrics, Carlsbad, CA, USA). Swabs saturated with saliva were placed within a SalivaBio Swab Storage Tube (Salivametrics, Carlsbad, CA, USA) and centrifuged to collect the saliva. Saliva samples were stored at −80 °C until viral DNA extraction.

Immediately prior to extraction, SM buffer (0.1 M NaCl, 50 mM Tris-HCl [pH 7.4]) was added to each saliva sample to obtain a final volume of 400 µL. Viral DNA was extracted from 200 µL of diluted sample using the High Pure Viral Nucleic Acid Kit (Roche Diagnostics, Indianapolis, IN, USA), and circular DNA in the extract was amplified using the Illustra TempliPhi Kit (GE Healthcare, Chicago, IL, USA). Illumina sequencing libraries were generated using the Illumina DNA Prep Kit (with Tagmentation) and sequenced on an Illumina HiSeq 2500 at Psomagen Inc. (Rockville, MD, USA).

Paired-end reads (2 × 150 bp) were trimmed using Trimmomatic-0.39 [54] and de novo assembled with MEGAHITv.1.2.9 [55]. After circular contigs were identified based on terminal redundancy, contigs >1000 nts were analyzed for viral-like sequences using Diamond [56] BLASTx against a local viral protein RefSeq database (release 220; downloaded September 2023). Potential PV-like contigs were confirmed using BLASTn [57]. Genomes were annotated using CenoteTaker2 [58] and refined with PaVE [59].

To determine the genera of the PV genomes characterized in this study, datasets of papillomavirus E1, E2, and L1 protein sequences were constructed using PaVE reference and non-reference sequences. The datasets were aligned with the *Varecia* PVs using MAFFT v7.113 [60] in AUTO mode. Alignments were trimmed with TrimAL [61] (0.2 gap threshold). ProtTest 3 [62] was used to determine the best-fit amino acid substitution models for each dataset. A partitioned maximum likelihood phylogenetic tree of concatenated E1 + E2 + L1 amino acid sequences was built using IQ-TREE 2 [63] with partition models LG + I + G for E1, LG + I + G + F for E2, and LG + I + G + F for L1. The tree was rooted with avian and reptilian PV sequences and edited in iTOL v6 [64].

Mitochondrial genomes were annotated using MITOS Web Server [65]. For mitochondrial genome comparisons, available mitochondrial genomes for lemur species within the *Lemuridae* family (and *Indriidae* as an outgroup) were aligned with mitochondrial genomes characterized in this study using MAFFT [60]. The mitochondrial genome maximum likelihood phylogenetic tree was built using IQ-TREE 2 with ModelFinder and ultrafast bootstrap (UFBoot) approximation (1000 bootstrap replicates) and edited in iTOL v6 [64].

## 3. Results

Our viral metagenomic workflows enabled the identification of three circular contigs, ranging in size from 7452 to 7770 nts in length, that represented complete PV genome sequences based on terminal redundancy. The mapped reads have been deposited at SRA under SRR26324874 and SRR26324875. The genome sequences are deposited in GenBank under accessions OR734654-OR734656. For OR734654 (7452 nts), the depth of coverage is 110× with 5463 reads, and for OR734655 (7770 nts) and OR734656 (7770 nts), the depth is 196× and 296× with 10134 and 15325 reads, respectively.

**Figure 2:**
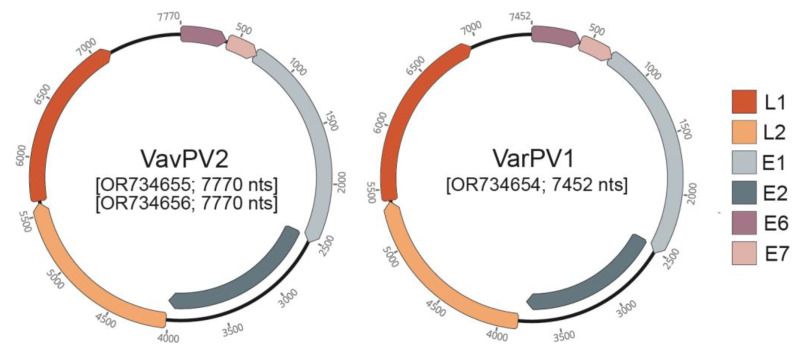
Annotations of complete PV genomes characterized from *V. rubra* saliva. Two of the complete genomes can be classified as VavPV2, a PV previously determined from *V. variegata* saliva. One of the complete genomes belongs to a new type and species and has been named Varecia rubra papillomavirus 1 (VarPV1).

All three genomes contain open reading frames for L1, L2, E1, E2, E6, and E7 (Figure 2). As PV-type demarcation is determined by L1 sequence similarity <70%, we compared L1 nucleotide identity between the *V. rubra*-derived papillomavirus L1 sequences characterized in this study and the previously characterized VavPV1 and VavPV2 L1 sequences detected from *V. variegata*.

OR734655 and OR734656 share 100% L1 nucleotide identity and were isolated from twin *V. rubra* lemurs M1 and J1, female lemurs housed together at the time of sampling. Further, OR734655 and OR734656 share 98% L1 nucleotide identity with VavPV2. Therefore, these two PVs belong to the same PV type (i.e., VavPV2). As VavPV2 has been found in two *V. rubra* and two *V. variegata* female individuals’ saliva from the Duke Lemur Center, this serves as a case of interspecies PV-type infection between non-human primate sister species.

The third complete PV genome sequence identified in *V. rubra*, OR734654, shares <68% L1 nucleotide identity with VavPV1, VavPV2, and all other PVs. Based on the PV species demarcation threshold of 70%, this PV represents a novel type which we have named Varecia rubra papillomavirus (VarPV) type 1. VarPV1 is the third PV type and species to be characterized in the lemuriform primates. Furthermore, VarPV1 was identified in the individual M1 from which we also identified VavPV2, thus suggesting an oral co-infection.

**Figure 3:**
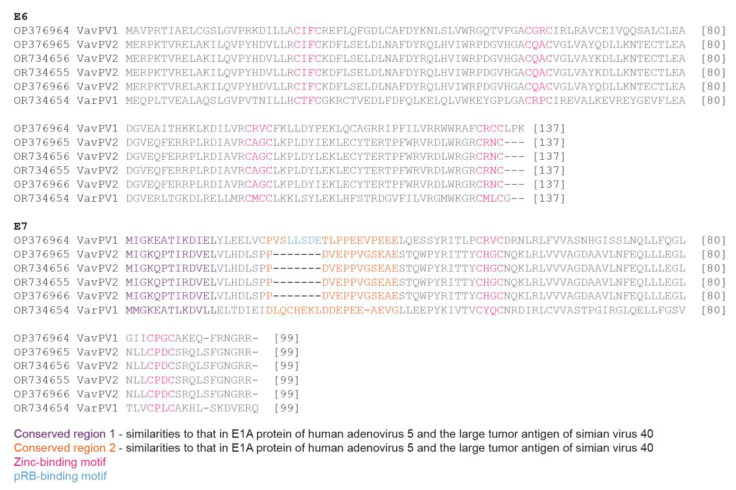
Summary of the conserved zinc-binding motifs (CxxC) in the E6 and E7 proteins, and conserved regions 1 and 2 and pRB-binding motif (Lx[C/S]xE) in the E7 protein of all lemur papillomaviruses.

In the E6 and E7 proteins of VarPV1 and VarPV2 from *V. rubra*, we identified the conserved zinc-binding domains (CxxC) (Figure 3). Unlike the pRB-binding motif (Lx[C/S]xE) identified in VavPV1 from *V. variegata*, we were unable to identify this motif in VarPV1 or VavPV2. We also identified the conserved regions 1 and 2 in VarPV1 and VavPV2 that are homologous to those in the E1A protein of human adenovirus 5 (family *Adenoviridae*) and the large tumor antigen of simian virus 40 (family *Polyomaviridae*) [66].

**Figure 4:**
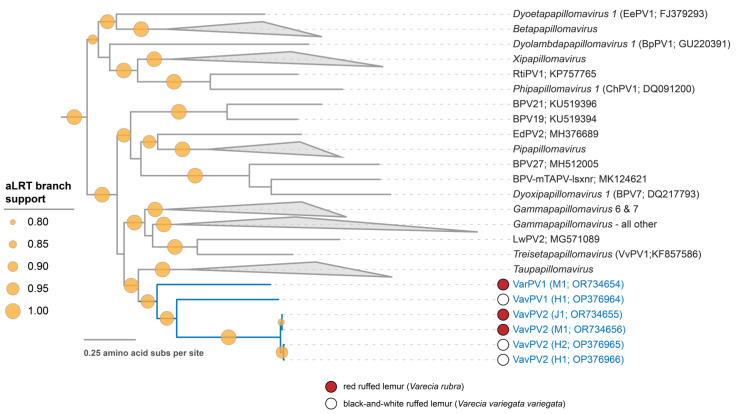
Partitioned maximum likelihood phylogenetic tree of concatenated amino acid sequences of E1, E2, and L1 showing the relationship of the lemur papillomaviruses with their nearest neighbors.

Based on their E1 + E2 + L1 protein sequence phylogeny, the two VavPVs and VarPV1 form a new cluster within a well-supported clade with sequences in the genera *Dyoxipapillomavirus, Gammapapillomavirus, Pipapillomavirus, Taupapillomavirus,* and *Treisetapapillomavirus*, in addition to six non-classified PV types, including BPV19, BPV21, and BPV27 (Figure 4). Overall, due to the divergence of the *Varecia* PV cluster from known PV genera, this new cluster likely represents at least one new genus.

**Figure 5:**
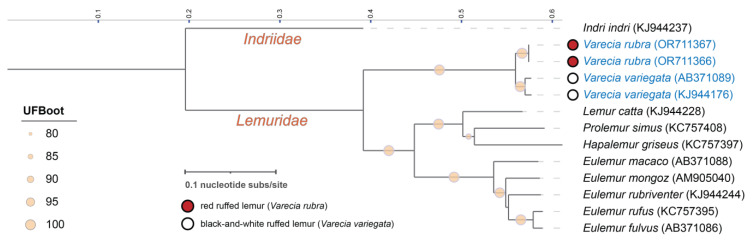
Maximum likelihood phylogenetic tree of host mitochondrial genomes. Only a subset of species within the *Lemuridae* family (and outgroup *Indriidae*) for which mitochondrial genomes were available are depicted. *V. rubra* mitochondrial genomes characterized in this study and publicly available *V. variegata* mitochondrial genomes are depicted in blue to allow for comparison. Branch support was determined with ultrafast bootstrap (UFBoot) approximation using IQ-TREE 2.

The results presented in this study highlight the use of viral metagenomics for determining the complete genomes of viruses potentially relevant to endangered species’ health. In addition, metagenomic protocols employing rolling circle amplification are also ideal for the determination of mitochondrial genomes. Two complete mitochondrial genomes were characterized in *V. rubra* saliva from individuals M1 and J1, which are female twins. The raw reads are deposited under SRA accessions SRR26324872 and SRR26324873, and genomes are deposited in GenBank under accessions OR711366 and OR711367. Both mitochondrial genomes are 16972 nt in length and share 100% nucleotide identity. The cytochrome-b (*cytb*) genes, the gene primarily used for phylogenetic species identification in lemurs, in both mitochondrial genomes are 1140 nt in length; the same length has been previously found in *V. rubra cytb* (accession number AY441450). Although sequences of *V. rubra* mitochondrial genome *cytb* and D-loops [67] have been published, there were no complete *V. rubra* mitochondrial genomes available on NCBI prior to this study. The complete mitochondrial genomes of *V. rubra* share 96.8% nucleotide identity with *V. variegata* (accession numbers AB371089 and KJ944176), emphasizing the close evolutionary relationship between the two species (Figure 5).

## 4. Discussion

Ongoing habitat loss has dramatically impacted *Varecia* populations in Madagascar. Additionally, given *Varecia*’s vital roles in primate evolutionary history and ecosystem services, large captive populations of around 800 *V. variegata* and 600 *V. rubra* are maintained globally [53,68]. To maintain healthy captive *Varecia* populations, understanding the pathogens that impact them is imperative for the survival of these species. The Duke Lemur Center in Durham (NC, USA) houses the largest and most diverse population of lemurs outside Madagascar and is currently home to 13 *V. variegata* and 12 *V. rubra* individuals. The Duke Lemur Center plays an active role in the Species Survival Plans and Population Analysis and Breeding and Transfer Plans for both *V. variegata* and *V. rubra.* Thus, the study of viruses circulating in captive populations is essential for learning about viral diversity within *Varecia* and implementing strategies to reduce the burden of pathogenic viruses that can be detrimental to their health and conservation.

Although the majority of PV infections are asymptomatic, information about PVs is relevant for lemur health as some types have been associated with invasive cancer in humans and non-human primates. In our previous work, we determined the first complete genomes of PVs in lemurs, VavPV1 and VavPV2, in *V. variegata* saliva. In this study, we present a case of interspecies infection by VavPV2 in *V. rubra*. In addition, a new PV type has been identified in *V. rubra* and termed VarPV1. In non-human primates, the two previously known instances of interspecies PV infection were between *Macaca mulatta* and *M. fascicularis,* and *Pan paniscus* and *P. troglodytes* (Figure 1). As non-human primate interspecies infection has only been detected in Old World monkeys and apes thus far, this study represents the first characterization of interspecies PV infection in strepsirrhines.

The *Varecia* PVs form a distinct cluster, likely representing at least one new genus, within a well-supported clade consisting of numerous established genera (Figure 4). In the genera *Dyoxipapillomavirus* and *Pipapillomavirus*, respectively, BPV7 and Phodopus sungorus papillomavirus (PsuPV)-1 types are members of this clade known to be capable of interspecies infection and tumor induction. BPV7 has been isolated from cutaneous papillomas in cattle [33], while PsuPV1 infection has been found to occasionally result in oral squamous cell carcinoma in hamsters [9]. Based on health exams completed by the Duke Lemur Center veterinary staff, the VavPVs and VarPV1 infections appear to be asymptomatic. Prior to this study, there were no instances of interspecies infection between non-human primates in the aforementioned clade, as MfPV3, MfPV8, MfPV11, MmPV1, and PpPV1 (Figure 1) all belong to the *Alphapapillomavirus* genus. This study expands our understanding of the diversity of PVs that can undergo interspecies infection in primate sister species.

Interspecies PV infection in non-human primates has thus far only been detected in evolutionarily closely related species within the same genus. *V. variegata* and *V. rubra* are closely related lemur species comprising the *Varecia* genus within the *Lemuridae* family. *V. rubra* was previously considered a subspecies of *V. variegata* until evidence supported its classification into a separate species, beginning in 2001 [69,70]. In Madagascar, the range of *V. rubra* is primarily restricted to the Masoala Peninsula in northeastern Madagascar, whereas that of *V. variegata* stretches through rainforest parcels from northeastern to southeastern Madagascar [52,53,71]. Providing evidence for their evolutionary relatedness, the geographic ranges of *V. variegata* and *V. rubra* have historically overlapped, resulting in reports of potential hybridization throughout time, based primarily on intermediate fur patterns, with hybridization likely to have been a rare occurrence [70,71]. *V. variegata* and *V. rubra* hybrids have also occurred in the past for the captive population studied, as *Varecia* were allowed to hybridize in the earlier history of the Duke Lemur Center, when it was known as the Duke University Primate Center, resulting in 53 hybrids (including 50/50, 7/8th, and 15/16th hybrids), 12 of which did not survive (stillborn or died shortly after birth). Although the M1 and J1 *V. rubra* individuals focused on for this study have no known level of hybridization in their pedigree, it is possible that relatives of M1 and J1 had interacted with hybrid individuals in the past. *V. rubra* and *V. variegata*’s close genetic relationship and exposure to one another through a captive environment are likely the drivers of the interspecies PV infection seen in this study.

Future work will delve more deeply into PV diversity across lemuriform primates through the sampling of additional host species, body sites (e.g., skin, anogenital region), and populations (i.e., wild versus captive). The same lemur individuals found to harbor PVs may be targeted for additional sampling across body regions to understand the cell tropism of VavPV1, VavPV2, and VarPV1.

## 5. Conclusions

In addition to expanding the known diversity of PVs, this study represents the first case of interspecies PV infection in strepsirrhines (VavPV2) and characterizes the third complete PV genome isolated from lemurs (VarPV1). The diversity of PVs characterized from two highly evolutionarily related lemur species, *V. variegata* and *V. rubra*, is likely just a glimpse into the undiscovered PV diversity circulating in over 100 species of lemurs. As *V. variegata* and *V. rubra* are critically endangered species with extensive global captive populations, understanding viral diversity in the *Varecia* genus is vital for the continued success of maintaining the health of captive populations and to provide a foundation for future comparisons to viruses found in wild *Varecia*. Lastly, the work here shows the value of viral metagenomics in recovering the complete genomes of viruses relevant to animal health, particularly for animals in which remarkably limited viral research has been conducted.

## Figures and Tables

**Table 1:** For each non-human primate (NHP) superfamily, the approximate number of extant NHP species is compared to the NHP species with PV sequences, including both partial sequences and complete genomes, available in NCBI. Additionally, the number of complete PV genomes available in NCBI for each primate superfamily is displayed. The data, obtained from NCBI Virus (https://www.ncbi.nlm.nih.gov/labs/virus/vssi/ (accessed on 18 November 2023)), emphasizes the scarcity of PV data across speciose NHP lineages. The six PV genomes available for lemurs include the three genomes characterized previously and the three genomes described in this study.

NHP Superfamily	Approx. Number of Extant Species	Species with PV Sequences (Partial and Complete) in NCBI	Number of Complete PV Genomes Available in NCBI
Ceboidea (New World Monkeys)	>100	*Alouatta caraya* *Alouatta guariba* *Callithrix penicillata* *Saimiri sciureus* *Sapajus* sp.	8
Cercopithecoidea (Old World Monkeys)	>130	*Colobus guereza* *Macaca fascicularis* *Macaca fuscata* *Macaca mulatta* *Papio hamadryas* *Piliocolobus tephrosceles*	23
Hominoidea (Apes, excludes humans)	~25	*Gorilla gorilla* *Pan paniscus* *Pan troglodytes*	4
Lemuroidea (Lemurs)	>100	*Varecia variegata* *Varecia rubra*	6
Lorisoidea (Lorisids & Galagos)	>25	-	0
Tarsioidea (Tarsiers)	>10	-	0

## Data Availability

The data for this study are available under BioProject PRJNA874427. The raw reads for this study are deposited under SRR26324872- SRR26324875. The papillomavirus genomes described in this study have been deposited in GenBank under accessions OR734654–OR734656. The mitochondrial genomes described in this study have been deposited in GenBank under accessions OR711366–OR711367.
